# A novel disulfidptosis and glycolysis related risk score signature for prediction of prognosis and ICI therapeutic responsiveness in colorectal cancer

**DOI:** 10.1038/s41598-023-40381-5

**Published:** 2023-08-16

**Authors:** Jiazheng Li, Chao Yang, Yongbin Zheng

**Affiliations:** Department of Gastrointestinal Surgery, Renmin Hospital of Wuhan University, Wuhan, China

**Keywords:** Cancer, Genetics

## Abstract

Disulfidptosis is a newly-identified non-programmed cell death mode with tight associations with glucose metabolism. Elevated glycolysis is an important metabolic feature of tumor cells, which fulfills the energy requirement for their rapid growth and progression. Our present study determined to develop a disulfidptosis and glycolysis related gene (DGRG) risk score signature to predict the prognosis and ICI therapeutic responsiveness for CRC patients. First, the gene expression and clinical profiles for CRC patients were obtained from TCGA and GEO database. Using weighted gene co-expression network analysis, we identified hub genes showing the strongest correlations with both disulfidptosis and glycolysis activities. Next, a DGRG risk score signature was successfully developed through univariate and least absolute shrinkage and selection operator method Cox regression method. A DGRG risk score-based nomogram could further enhance the predictive performance. In addition, an array of systemic analysis was performed to unravel the correlation of DGRG risk score with tumor microenvironment. The results showed that CRC patients with low DGRG risk level had up-regulated immune cell infiltrations, enhanced metabolic activities and heightened gene mutation frequencies, while high risk patients was the opposite. Moreover, our present study identified low risk CRC patients as potential beneficiaries from immune checkpoint inhibitor (ICI) therapies. Our present work highlighted the potential utility of DGRG risk score signature in prognosis prediction and ICI responsiveness determination for CRC patients, which demonstrated promising clinical application value.

## Introduction

Colorectal cancer (CRC) is one of the most common malignancies in digestive system with high incidence and mortality rates. Globally, CRC accounts for the third and second positions for newly-emergent cases and cancer-related deaths, respectively^1^. In recent years, immunotherapy represents an emerging paradigm for the comprehensive management of cancer and have achieved effective therapeutic responsiveness in many types of malignant tumors^2–5^. For CRC patients, the potential utility of immune checkpoint inhibitors (ICI) as a novel therapeutic strategy is gaining widespread attention. So far, nivolumab monotherapy and the combined utility of nivolumab with ipilimumab was confirmed to induce optimistic and durable treatment responses by Checkmate-142 phase II clinical trials^6,7^. The two drugs were approved by FDA for the treatment of metastatic CRC patients with microsatellite instability status in 2018^8^. Of note, efficient and durable disease control in response to ICI therapies is impacted by multiple external and internal factors, among which the heterogeneity in tumor microenvironment (TME) is regarded as a key determinant^9^. Patients with abundant immune cell infiltration and sufficiently-activated anti-tumor immune response encompassed a “hot tumor” TME and were associated with favorable therapeutic sensitivity towards ICI treatment. On the contrary, an immune-desert or “cold tumor” TME often represents impaired therapeutic responses^9^. Therefore, a deeper understanding about the potential contributors to TME heterogeneity is of great importance to find out the potential beneficiaries of ICI treatment and improve the survival outcomes for CRC patients.

Recently, a group of scientists identify a novel programmed cell death (PCD) mode induced by aberrant accumulation of intracellular disulfides and define it as disulfidptosis^10^. Mechanistically, SLC7A11 high expression coupled with glucose depletion causes the excessive disulfide bond formation among cytoskeleton proteins, which further leads to the collapse of actin network and cell death^10^. Of note, the pharmacological blockade of glucose uptake by GLUT inhibitors was proved to exert cancer killing effects by the promoting disulfidptosis in SLC7A11-high tumor cells, highlighting the therapeutic utility of disulfidptosis-induction strategies in cancer treatment^10^. However, since the conceptualization of disulfidptosis is in its infancy, the potential regulatory role of this PCD pattern in tumorigenesis remains largely unknown and deserves further investigation.

Metabolism reprogramming, defined as the significant alterations in metabolic pathways in tumors in comparison to normal tissues, represents one of the hallmarks of cancer^11,12^. In terms of glucose metabolism, cancer cells rely heavily on glycolysis to meet the energy requirements and elevated glycolysis could be observed even in oxygen-replete conditions. The unique aerobic oxidation mode, known as Warburg effect, engenders cancer cells with enhanced proliferative and metastasis abilities, and is tightly associated with recurrence and multidrug resistance^13,14^.

Considering the intimate association between disulfidptosis and glucose metabolism, as well as the essential role of glycolysis in tumor cells’ metabolic process, we determined to explore the potential interaction between disulfidptosis—and glycolysis-related genes (DGRG) in the pathogenesis of CRC. In the present study, we first screened out hub genes in association with disulfidptosis and glycolysis through weighted gene co-expression network analysis (WGCNA) in two CRC patients cohorts. Then, using univariate and Least Absolute Shrinkage and Selection Operator (LASSO) Cox regression analyses, we developed DGRG risk score signature, and later confirmed its favorable performance in prognosis prediction. In addition, the association of DGRG risk score with TME landscape and the potential therapeutic utility of it in guiding ICI therapies were further investigated.

## Results

### Characterization of disulfidptosis-related genes in CRC

We first investigated the expression landscape and clinical relevance of 29 disulfidptosis-related genes. In TCGA cohort, 22 of the 29 genes were differentially expressed between CRC tumor and normal tissues (Fig. S2A). According to Pearson correlation analysis, there were extensive and strong correlations within these genes in terms of the expression levels (Fig. S2B). In addition, we integrated the clinical data in TCGA and GEO cohort and assessed the prognostic value of disulfidptosis-related genes using Kaplan–Meier method. The result identified FLNA as a risk factor for worse survival outcomes, while OXSM as a protective factor for improved prognosis (Fig. S2C,D). Furthermore, a total of ten genes demonstrated intimate associations with clinical stages, including CD2AP, TLN1, FLNA, RAC1, BRK1, ABI2, GYS1, NDUFS1, OXSM and RPN1 (Fig. S2E). Therefore, our analysis revealed the expression landscape and clinical relevance of disulfidptosis-related genes, highlighting the potential role of disulfidptosis in CRC tumorigenesis.

### Disulfidptosis and glycolysis activities showed positive correlations in CRC

**Figure 1:**
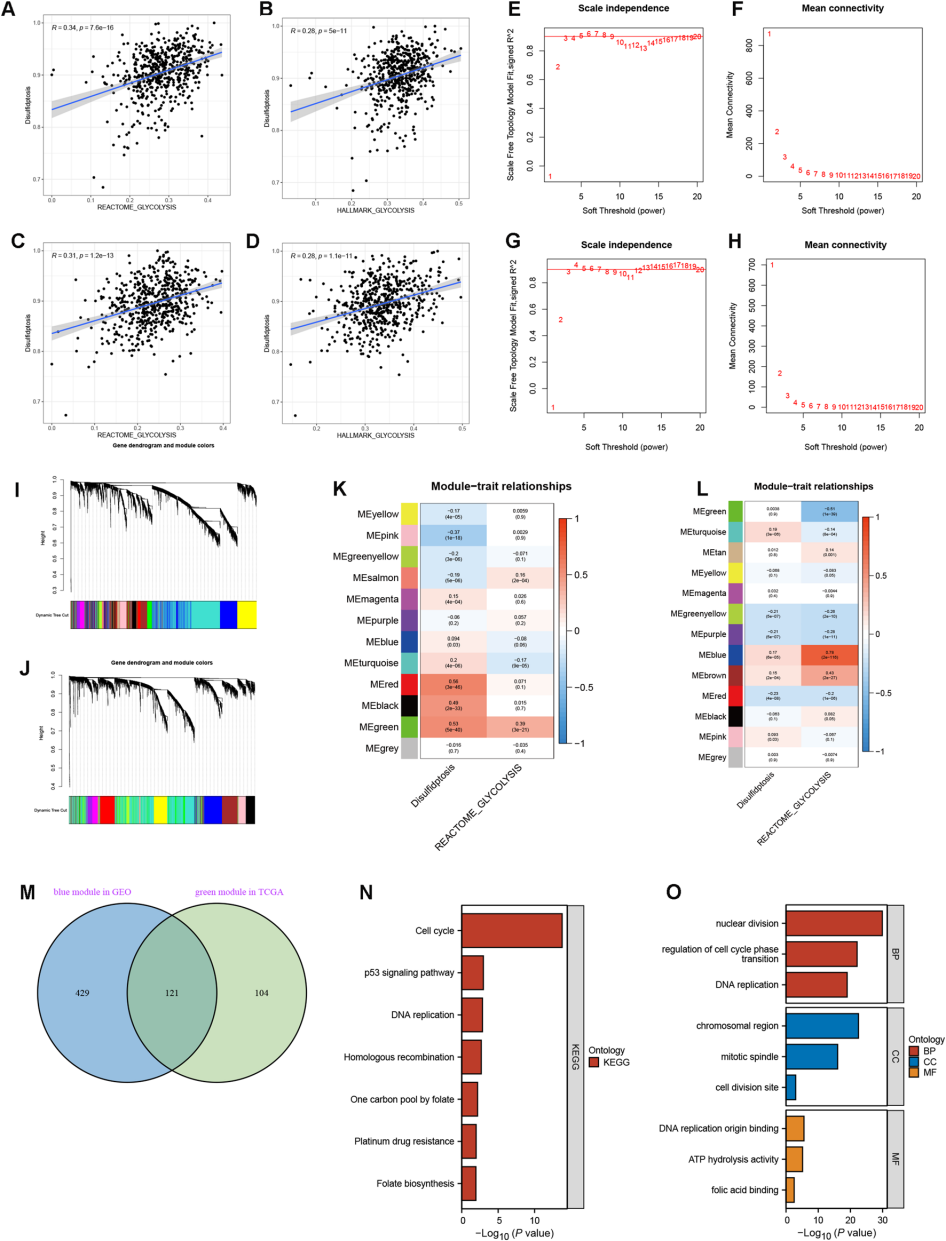
Identification of hub genes by WGCNA. (**A–D**) Correlations between the disulfidptosis enrichment scores with Hallmark and Reactome glycolysis enrichment scores in (**A**,**B**) TCGA and (**C**,**D**) GEO cohort. (**E–H**) Analysis of scale independence and mean connectivity to determine the optimal soft-thresholding power β in (**E**,**F**) TCGA and (**G**,**H**) GEO cohort. (**I**,**J**) Dynamic tree cutting algorithms for identification of genes with similar expression patterns in (**I)** TCGA and (**J)** GEO cohort. Grey module involves genes that can’t be classified into any other module. (**K**,**L**) Correlations of the eigengene in each module with disulfidptosis and glycolysis enrichment scores in (**K**) TCGA and (**L**) GEO cohort. (**M**) Venn diagram showing the overlapping hub genes between TCGA green module and GEO blue module. (**N**) KEGG pathway analysis for hub genes. (**O**) GO biological function analysis for hub genes. *BP* biological process, *CC* cellular compartment, *MF* molecular function.

We estimated the enrichment scores of disulfidptosis and glycolysis using ssGSEA method and investigated their associations via Spearman correlation analysis. As shown in Fig. 1A–D, for both TCGA and GEO cohort, the enrichment scores of disulfidptosis were positively correlated with that of glycolysis estimated by both Reactome and Hallmark gene sets, suggesting that cancer cells with enhanced glycolytic activity were more susceptible to disulfidptosis. Presumably, high consumption rate of glucose caused by enhanced glycolytic activity renders a glucose-deficient environment^15^, which is supposed to be conducive for induction of disulfidptosis.

### Identification of hub genes by WGCNA

In view of the intimate associations between disulfidptosis and glycolysis activities, we aimed to find out hub genes co-correlated with the two processes (DGRGs) using WGCNA. To construct a scale-free topology network, β was set as 6 and 5 for TCGA (Fig. 1E,F) and GEO cohort (Fig. 1G,H), respectively. Subsequent dynamic tree cutting algorithm identified 12 and 13 gene modules in TCGA (Fig. 1I) and GEO (Fig. 1J) cohort, respectively. Based on Pearson correlation analysis, the green module for TCGA cohort and the blue module for GEO cohort showed the strongest correlations with both disulfidptosis and glycolysis activities (Fig. 1K,L). Genes involved in the two modules were listed in Table S3. Subsequent intersection analysis identified 121 overlapping hub genes between the two modules (Fig. 1M). According to KEGG pathway and GO biological function analysis, these hub genes were mainly involved in cell division and DNA replication activities, suggesting their potential regulatory role in cell proliferation. Of note, “p53 signaling pathway” and “platinum drug resistance” also showed certain degrees of enrichment, which was indicative of the correlations of hub genes with cancer development. Besides, our analysis also correlated these hub genes were with metabolic activities (e.g. one carbon pool by folate ATP hydrolysis activity) (Fig. 1N,O).

### Construction and validation of DGRG risk score signature

**Figure 2:**
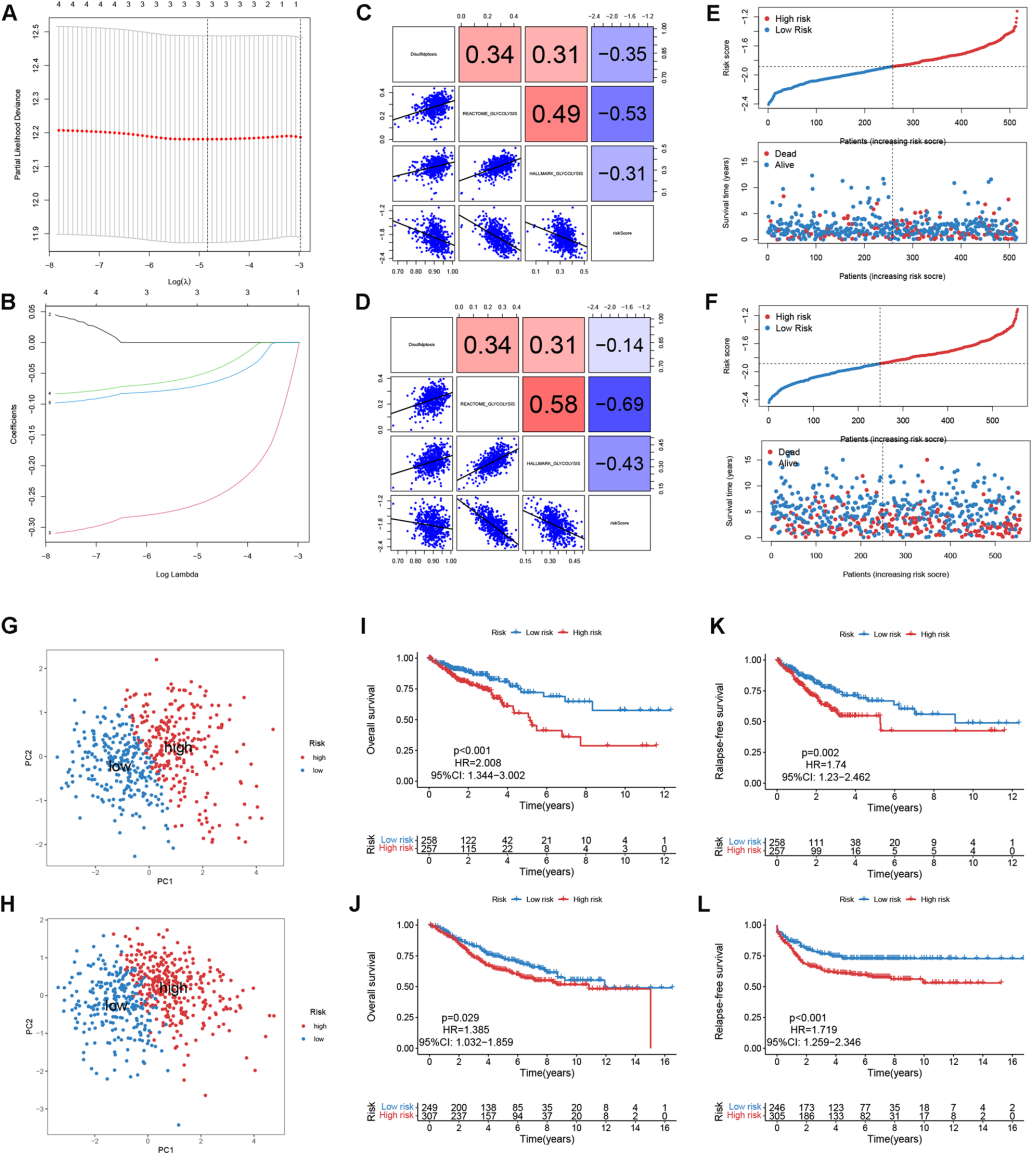
Construction and validation of DGRG risk score signature. (**A**) LASSO Cox regression analysis identifies the optimal tuning parameter. (**B**) LASSO coefficients for prognosis-related hub genes. (**C**,**D**) Correlations of DGRG risk score with disulfidptosis and glycolysis enrichment scores for (**C**) TCGA and (**D)** GEO patients. (**E**,**F**) DGRG risk score’s distribution and its correlation with survival status in (**E)** TCGA and (**F**) GEO cohort. (**G**,**H**) PCA analysis for high and low risk patients in (**G**) TCGA and (**H**) GEO cohort. (**I**,**J**) Kaplan–Meier plots showing the differences in OS between high and low risk patients in (**I**) TCGA and (**J)** GEO cohort. (**K**,**L**) Kaplan–Meier plots showing the differences in RFS between high and low risk patients in (**K**) TCGA and (**L**) GEO cohort. *HR* hazard ratio, *CI* confidence interval.

To assess the prognostic value of hub genes and develop a quantitative measurement for patients’ risk level, we first performed univariate Cox regression analysis for TCGA patients and identified five genes closely related to OS (Fig. S3), followed by LASSO Cox regression analysis (Fig. 2A,B), which finally screened out three eligible genes including ORC1, DHFR and HMMR. Based on the coefficients of LASSO Cox regression analysis, the DGRG risk score was calculated as follows: DGRG risk score = (− 0.257819* expression of ORC1) + (− 0.049340* expression of DHFR) + (− 0.067609 * expression of HMMR).

Of note, the risk score was inversely correlated with the enrichment scores of dusulfidptosis and glycolysis (Spearman correlation analysis, Fig. 2C). Subsequently, CRC patients in TCGA cohort were divided into high and low risk groups according to the median risk score. Risk score’s distribution and its correlation with patients’s survival status were depicted in Fig. 2E. According to PCA analysis, there were remarkable deviations in signature gene expression patterns between two risk groups, indicating the robustness of the DGRG-based risk classification for CRC patients (Fig. 2G). Moreover, Kaplan–Meier analysis correlated low risk patients with significant advantages in both overall survival (OS, log-rank p < 0.001, Fig. 2I) and relapse-free survival (RSF, log-rank p = 0.002, Fig. 2K).

Thereafter, the DGRS risk score of each GEO patient was estimated using the same formula. It was found that risk score also had negative associations with both dusulfidptosis and glycolysis activities (Fig. 2D). Then GEO patients were also classified into different risk groups following the same criterion (Fig. 2F). PCA analysis corroborated the robustness of risk level classification (Fig. 2H). Finally, the survival advantages could also be observed in GEO patients with low risk level (OS, log-rank p = 0.029, Fig. 2J; RSF, log-rank p < 0.001, Fig. 2L).

### Clinical correlation analysis

**Figure 3:**
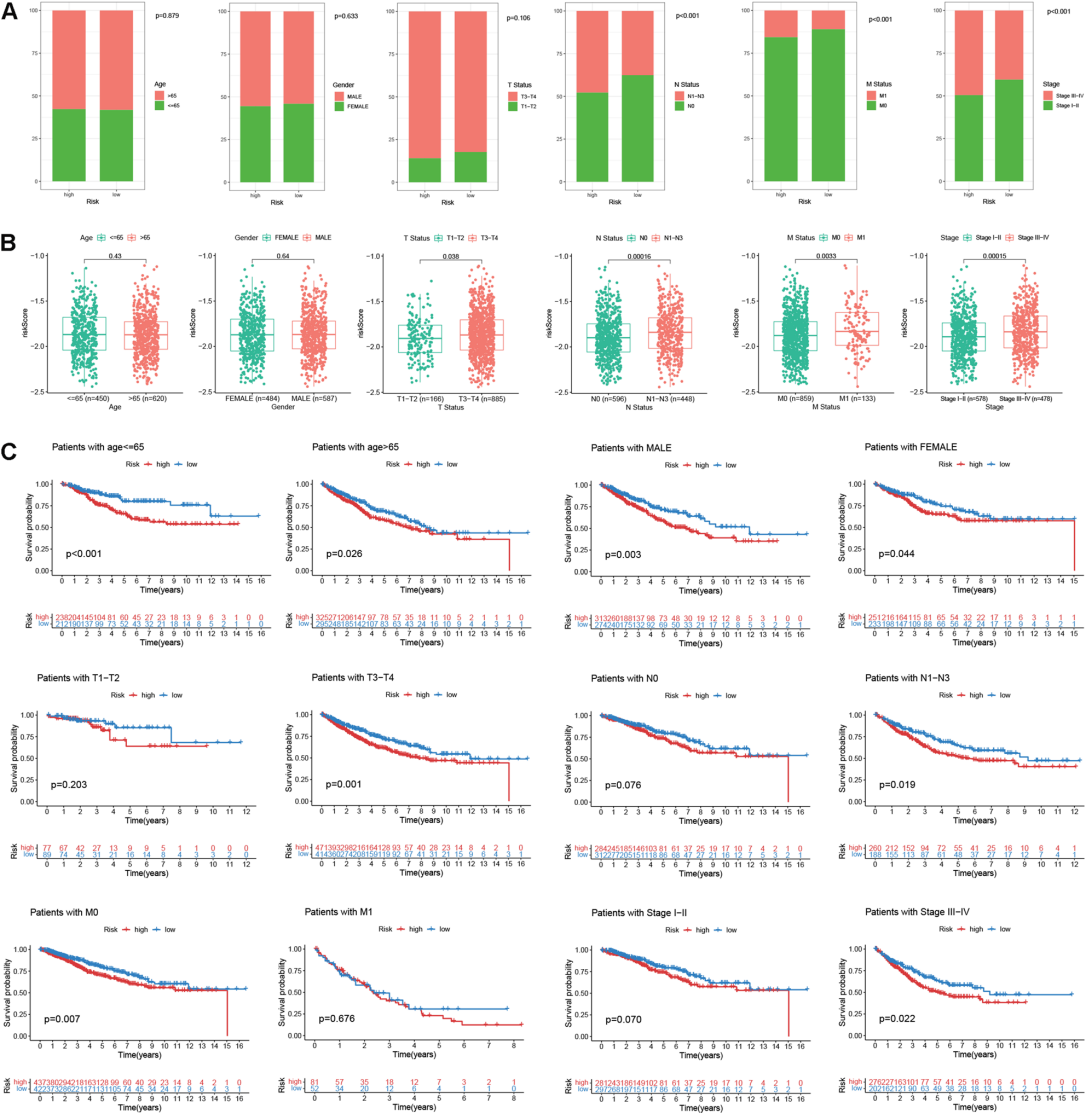
Clinical correlation analysis and nomogram construction. (**A**) Distributions of patients with different age, gender, T, N, M status and tumor stage in high and low risk groups. (**B**) Risk score differences for patients with different age, gender, T, N, M status and tumor stage. (**C**) Kaplan–Meier plots for high and low risk patients in different clinical subgroups stratified by age, gender, T, N, M status and tumor stage.

To assess the clinical implication value of DGRG risk score, we merged CRC patients in both TCGA and GEO cohorts and divided them into multiple subgroups according to age, gender, T, N, M status and tumor stage. It was suggested that lymph node metastasis (N1-N3 status), distant metastasis (M1 status) and advanced tumor stages (stage III-IV) were more frequently happened in high risk patients compared with the low risk counterpart (Fig. 3A). Analogously, patients with progressive clinical traits also demonstrated enhanced risk scores (Fig. 3B). DGRG risk score signature also had favorable performance in differentiating CRC patients’ survival regardless of their age and gender. However, for CRC patients with T1-T2 status, N0 status, M1 status, as well as stage I-II disease, there were no significant differences in OS between high and low risk groups (Fig. 3C).

### Nomogram construction

**Figure 4:**
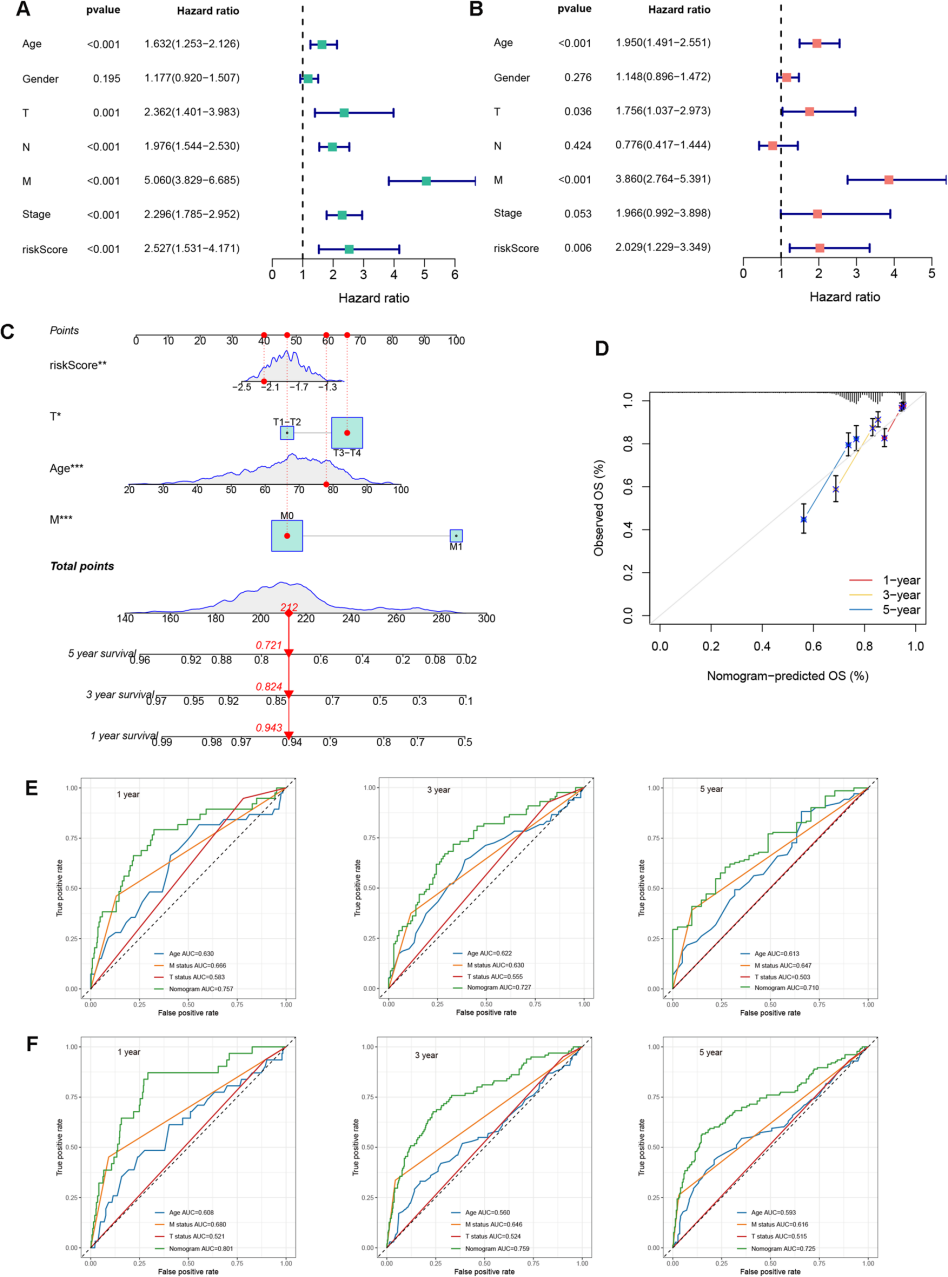
Nomogram construction. (**A**,**B**) Univariate and multivariate Cox regression analysis for DGRG risk score and clinical traits including age, gender, T, N, M status and tumor stage. (**C**) Nomogram integrating DGRG risk score, age, T and M status for prediction of 1, 3 and 5 year survival probabilities. (**D**) Calibration curve of nomogram. (**E**,**F**) ROC analysis comparing the performance of nomogram, age, T and M status for prediction of 1, 3 and 5 year survival probabilities in (**E**) TCGA and (**F**) GEO cohort.

To enhance the clinical utility of DGRG risk score model, we proposed to develop a nomogram that integrated the risk score and other prognosis-related clinical factors. We first performed univariate and multivariate Cox analysis to comprehensively evaluate the prognosticator value of all variables. After adjusting for age, gender, T, N, M status and tumor stage, the association of DGRG risk score with patients’ survival remained statistically significant (Fig. 4A,B), suggesting that DGRG risk score was an independent risk factor for worse prognosis. Subsequently, a nomogram integrating DGRG risk score and other prognosis-related variables revealed by multivariate Cox analysis including age, T and M status was constructed to predict the survival probabilities of CRC patients at different time points (Fig. 4C). For an individual CRC patients, when knowing the exact value of a variable, users could find its weighted point by projecting it to the points bar on the top. The total points could be obtained by adding up all the weighted points together. Then the survival probabilities at 1, 3 and 5 year for this patient could be speculated according to the three scale bars at the bottom by projecting the total point downwards.

According to the calibration curve, there were satisfactory agreements between the nomogram forecasts and the actual observations (Fig. 4D). For both TCGA and GEO patients, AUCs of nomogram for predicting 1, 3 and 5 year OS were all greater than 0.700. Besides, predictions of 1, 3 and 5 year OS using nomogram had superiorities over the age, T and M status-only models (Fig. 4E for TCGA cohort, Fig. 4F for GEO cohort).

### Functional enrichment analysis

**Figure 5:**
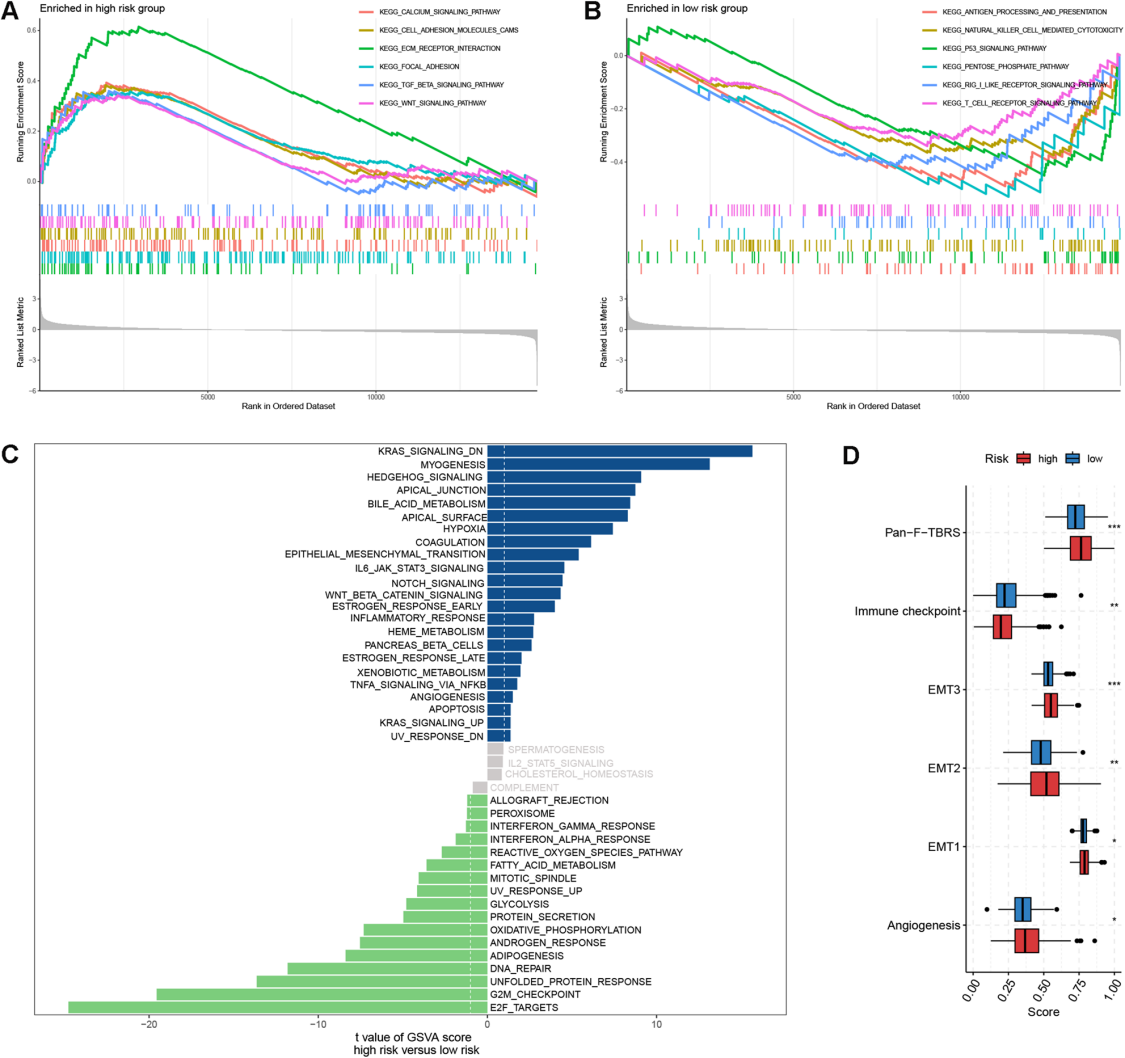
Functional enrichment analysis. (**A**,**B**) GSEA analysis for two risk groups. (**C**) GSVA analysis based on Hallmark gene set for two risk groups. (**D**) Differences in enrichment scores of Mariathasan gene set between two risk groups. *EMT* epithelial-mesenchymal transition, *pan*-*F*-*TBRS* pan-fibroblast TGFβ. Statistical significance: *p < 0.05; **p < 0.01; ***p < 0.001.

**Figure 6:**
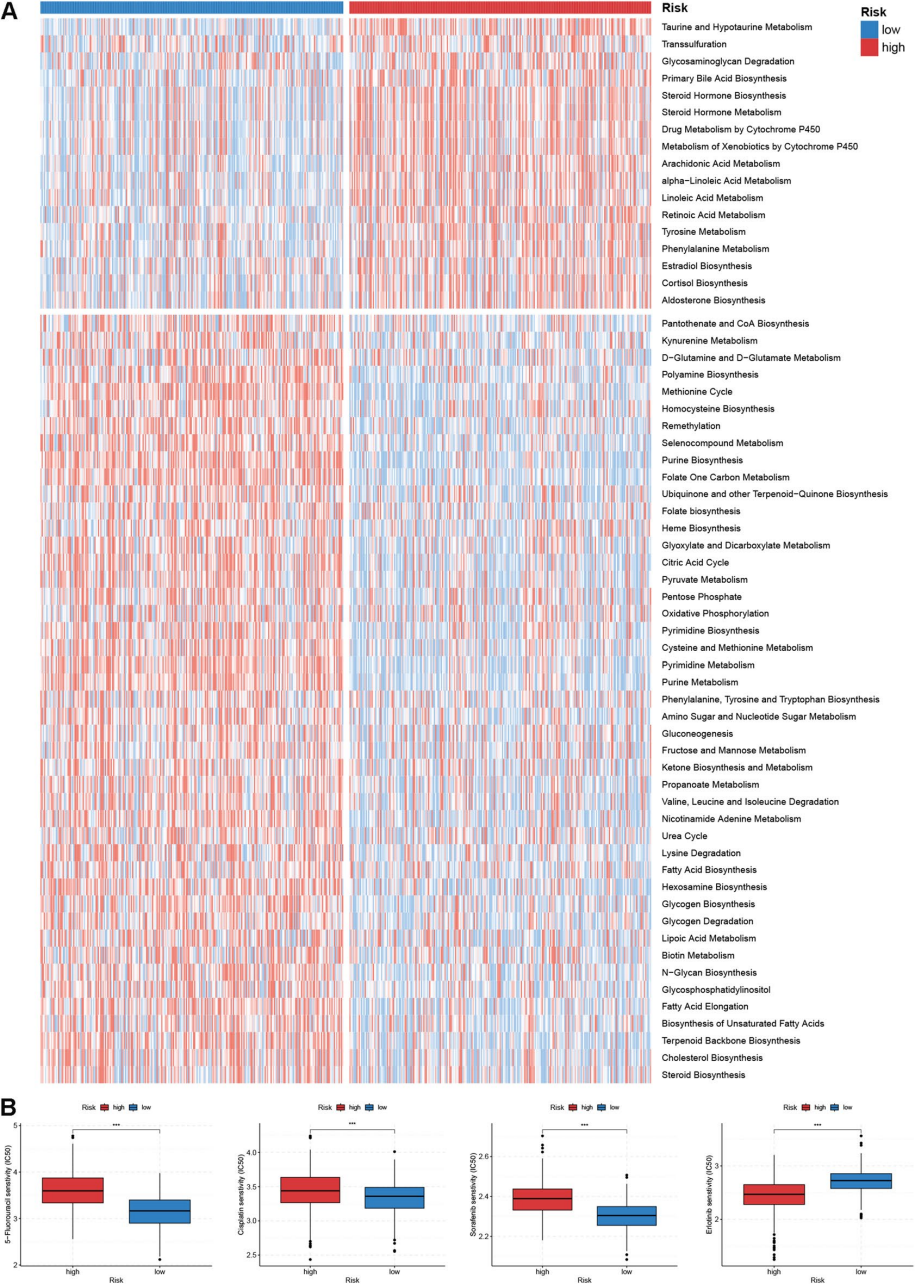
Metabolism and drug sensitivity analysis. (**A**) Heatmap showing metabolism enrichment patterns for two risk groups. (**B**) Drug sensitivity analysis of 5-flurouracil, cisplatin, sorafenib and erlotinib for high and low risk patients.

To shed light on the enrichment patterns of biological functions in the two risk group, we first performed GSEA analysis based on KEGG gene set. As shown in Fig. 5A and B, carcinogenic pathways such as focal adhesion, Wnt signaling pathway and TGF beta signaling pathway, were markedly enriched in the high risk group; whereas pathways correlated with immune activities and glucose metabolism, such as T cell receptor signaling pathway, NK cell receptor pathway and pentose phosphate pathway had elevated enrichment levels in the low risk group. Similarly, GSVA analysis using Hallmark gene set also observed upregulation of carcinogenic processes (e.g. Kras signaling, TNFα signaling via NFκB, Wnt beta catenin signaling, etc.) in the high risk group. While low risk patients were more implicated in signatures correlated with immune response (e.g. interferon alpha and gamma response, allograft rejection), cell cycle (e.g. G2M checkpoint, E2F target, DNA repair) and metabolism (e.g. glycolysis, adipogenesis, oxidative phosphorylation) (Fig. 5C). Notably, some stroma-related signatures including hypoxia, angiogenesis and epithelial-mesenchymal transition (EMT), were all significantly enriched in the high group. For Mariathasan gene signature, high risk patients had elevated enrichment scores of stroma-related items including angiogenesis, EMT1-3 and pan-fibroblast TGFβ response signature (pan-F-TBRS), while low risk patients had increased enrichment scores for immune checkpoint (Fig. 5D). Furthermore, the quantification of metabolic processes revealed that low risk patients showed generally stronger metabolic activities, especially for nutrient metabolism including glucose, lipid and amino acids; while low risk patients were more concerned with metabolism for steroid hormone, organic acids and drugs (Fig. 6A).

Taken together, functional enrichment analysis correlated high risk patients with up-regulated carcinogenic and stromal activities, whereas low risk patients with enhanced immune and metabolic activities.

### Immune infiltration and anti-tumor immunity analysis

**Figure 7:**
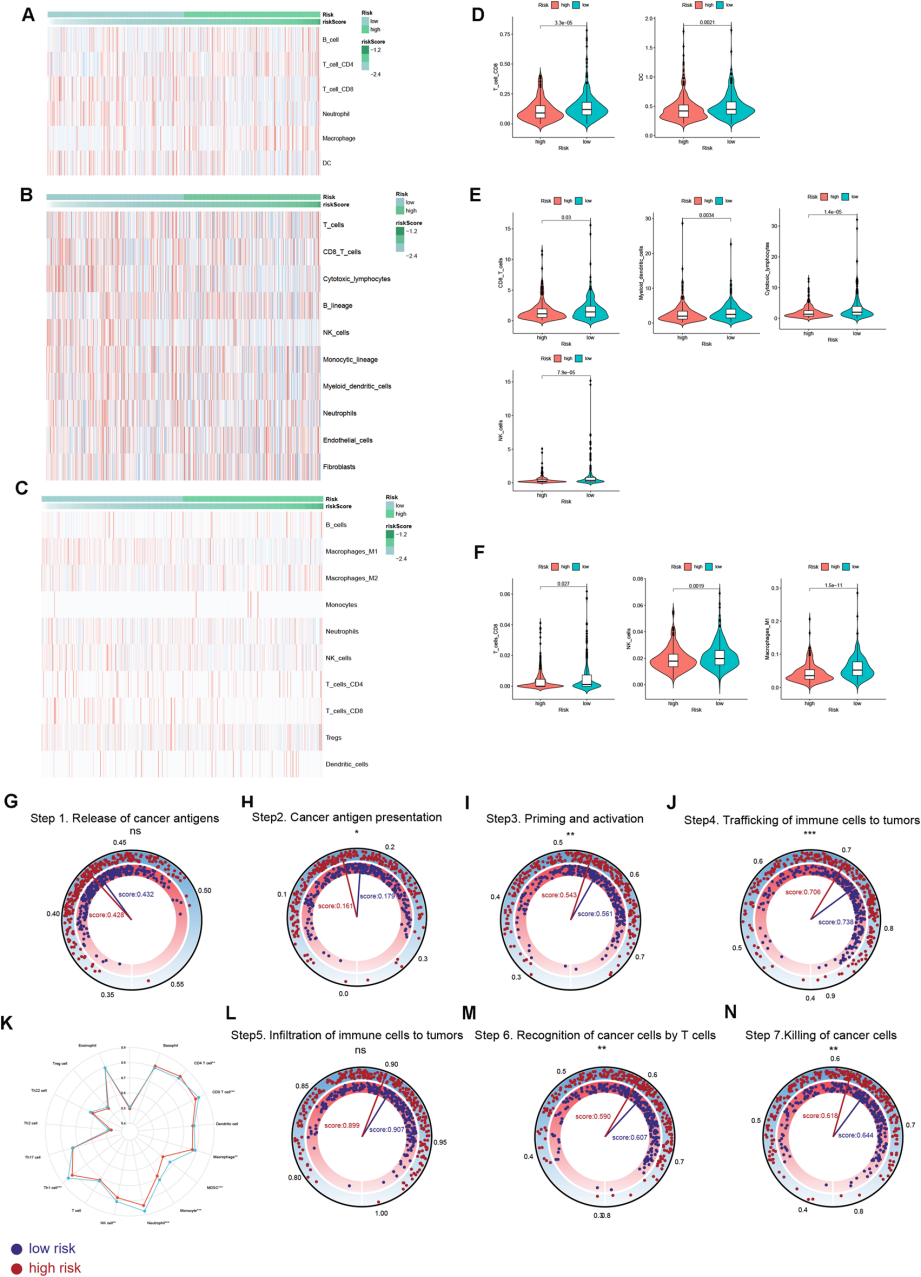
Immune infiltration and anti-tumor immunity analysis. (**A-C**) Heatmaps for the immune cell infiltration landscapes in two risk groups. (**D-F**) Differences in immune cell infiltration scores between high and low risk patients. Immune cell infiltration scores were estimated by (**A**,**D**) TIMER, (**B**,**E**) MCPcounter and (**C**,**F**) QUANTISEQ. **(G-N**) Analysis of the activation scores of seven-step anti-cancer immunity cycle for high and low risk patients. (**G**) Difference in the ability of cancer antigen releasing (step 1) between two risk groups. (**H**) Difference in the activity of cancer antigen presentation (step 2) between two risk groups. (**I**) Difference in the ability of priming and activating immune cells (step 3) between two risk groups. (**J**) Difference in the overall ability of trafficking immune cells to tumors (step 4) between two risk groups. (**K**) Difference in the ability of trafficking different immune cell subtypes to tumors between two risk groups. (**L**) Difference in the degrees of immune cell infiltrations (step 5) between two risk groups. (**M**) Difference in the T cell’s recognition ability for cancer cells (step 6) between two risk groups. (**N**) Difference in the ability of killing cancer cells (step 7) between two risk groups. Statistical Significance: *p < 0.05; **p < 0.01; ***p < 0.001; *ns* not significant.

We estimated the immune cell infiltration scores in two risk groups by TIMER, MCPcounter and QUANTISEQ algorithms. Heatmaps in Fig. 7A–C depicted distribution of the infiltration scores in two risk groups. Of note, anti-cancer immune cells including CD8 T cells, cytotoxic lymphocytes (CTLs), NK cells, and dendritic cells (DCs) and M1 macrophages were all densely infiltrated in the TME of low risk patients (Fig. 7D–F). Comparisons of the activity score of the seven-step anti-tumor immunity between two risk groups were shown in Fig. 7G–N. It was revealed that the activities of low risk patients for cancer antigen presentation (step 2), priming and activating immune cells (step 3), T cells’ recognition of cancer cells (step 6) and killing of cancer cells (step 7) were all stronger than that of high risk patients. The overall ability of trafficking immune cells to tumor sites (step 4) was stronger for patients with low risk (Fig. 7J). Specifically, Low risk patients showed stronger trafficking abilities towards various immune cell subtypes in comparison to high risk counterpart (Fig. 7K). Taken together, the above analysis correlated low risk patients with a “hot tumor” phenotype characterized by increased anti-tumor immune cell infiltrations and elevated activation status of anti-tumor immunity.

### ICI therapeutic response analysis

**Figure 8:**
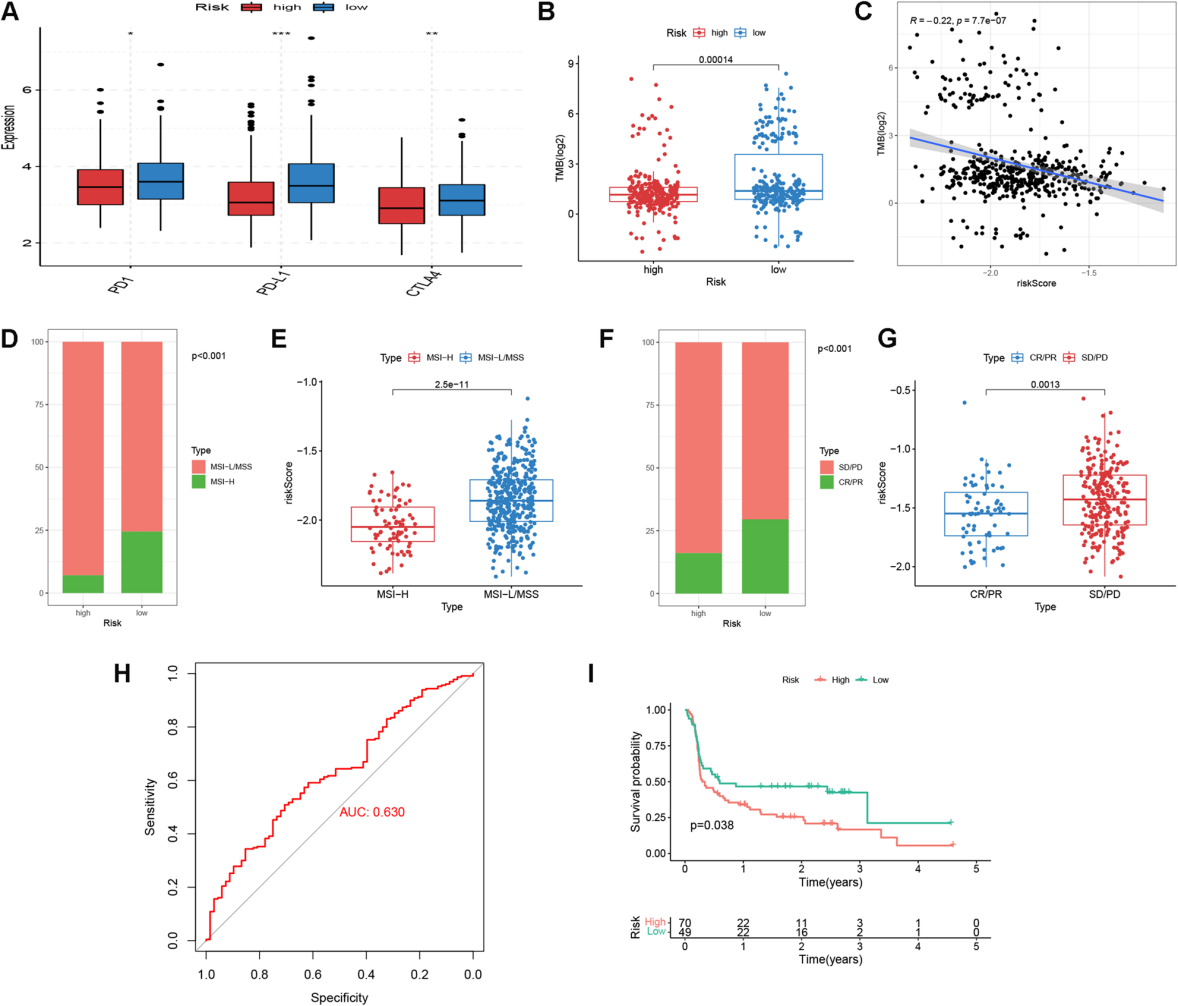
ICI sensitivity analysis. (**A**) Differences in PD1, PD-L1 and CTLA4 expressions between high and low risk patients. (**B**) TMB differences between high and low risk patients. (**C**) Spearman correlation analysis for TMB and risk score. (**D**) Distribution of MSI status in two risk groups. (**E**) Risk score differences between patients with MSI-H and MSI-L/MSS. *MSI-H* microsatellite instability-high, *MSI-L/MSS* microsatellite instability-low/microsatellite stable. (**F**) Responsiveness to ICI treatment in two risk groups for IMvigor210 cohort. *SD/PD* stable disease/progressive disease, *CR/PR* complete response/partial response. (**G**) Risk score differences between patients with SD/PD and CR/PR in IMvigor210 cohort. (**H**) ROC analysis of risk score for prediction of ICI responsiveness in IMvigor210 cohort. (**I**) Kaplan–Meier plots of progression-free survival for high and low risk patients in David Liu’s cohort.

The above analysis suggested that patients with low risk demonstrated a “hot tumor” phenotype, we then asked whether this group of patients responded better to ICI therapies. To tackle with this, we analyzed the differences in immune checkpoint gene expressions, TMB value and MSI status between high and low risk patients. Our results suggested that low risk patients exhibited upregulations of PD1, PD-L1 and CTLA4 (Fig. 8A), as well as higher TMB values (Fig. 8B). Besides, the risk score was demonstrated to be inversely correlated with TMB (Spearman correlation coefficient = − 0.22, p = 7.7 e^-7^, Fig. 8C). For MSI status, there were larger portions of patients with MSI-H status being classified into the low risk group (Fig. 8D). Analogously, MSI-H patients also trended towards lower risk scores (Fig. 8E). Then, the DGRG risk score signature was applied to two external ICI cohorts to verify the performance in predicting ICI sensitivity. In IMvigor210, patients acquiring complete or partial response (CR/PR) accounted for a higher proportion in the low risk group and were associated with reduced risk scores (Fig. 8F,G). ROC analysis confirmed the predictive efficacy of risk score in discriminating responders to ICI treatment, (AUC = 0.630, Fig. 8H). For David Liu’s cohort, the progression-free survival of low risk patients were better than that of high risk patients (log rank p = 0.038, Fig. 8I). Furthermore, we performed IHC experiment to examine the protein expressions of model gene ORC1and immune checkpoint gene PD-L1 in 10 CRC FFPE samples. It was suggested that expression of ORC1 showed strong and positive correlation with immune checkpoint gene PD-L1 (Fig. S4B). Representative IHC images from two patients were shown in Fig. S4A. Since ORC1 contributed the biggest part to the DGRG risk score, and CRC patients with up-regulated ORC1 expression were supposed to demonstrate reduced DGRG risk scores, the present IHC results to some extent confirmed the up-regulated PD-L1 expression and therefore the potentially improved ICI therapeutic sensitivity for low risk patients, which was in consistency with the above bioinformatic analysis results.

### Gene mutation analysis

**Figure 9:**
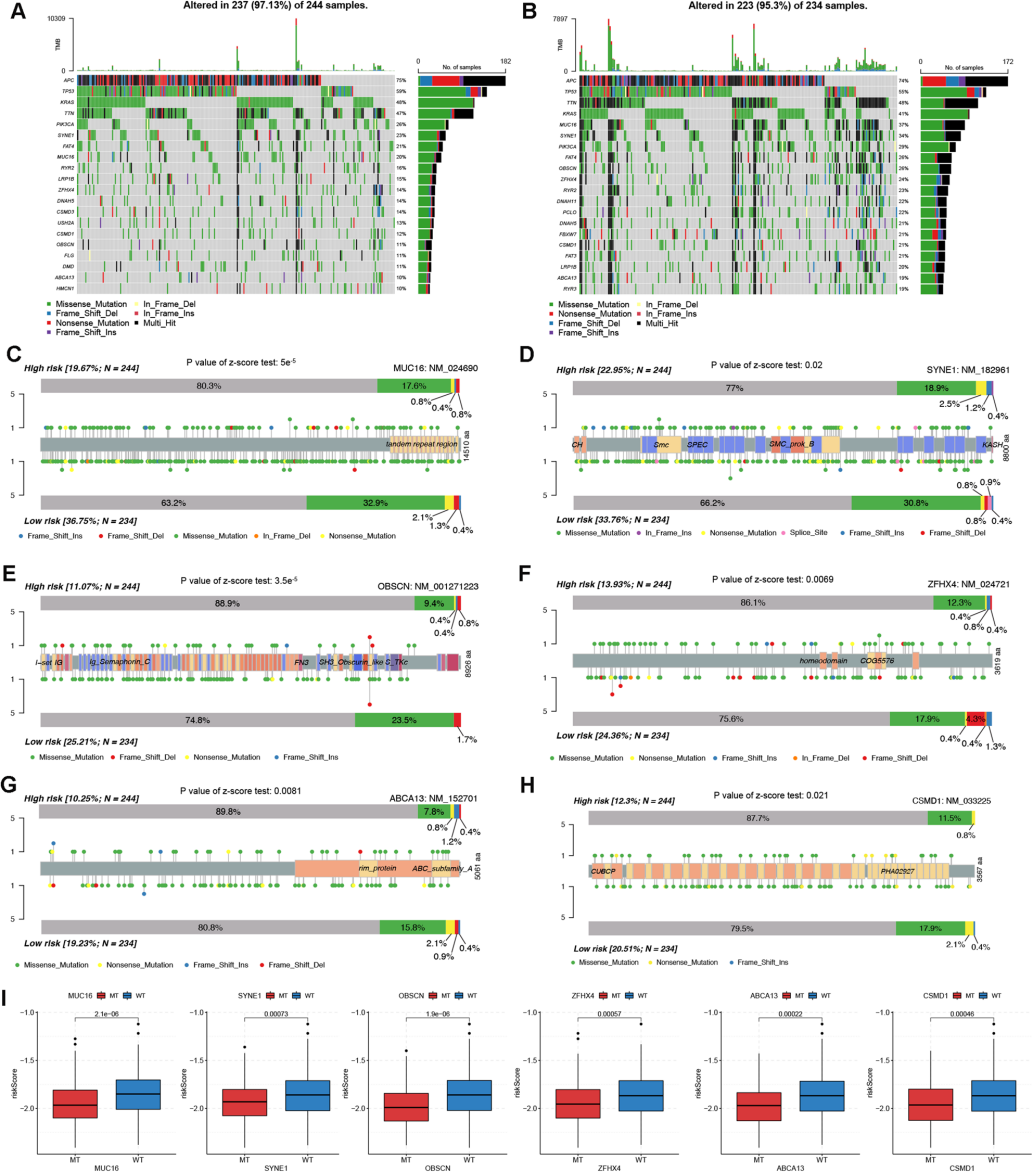
Gene mutation analysis. (**A**,**B**) Waterfall plots depicting gene mutation landscape of the top 20 most frequently mutated genes in high (**A**) and low (**B)** risk groups. Each column represents an individual patient. The percentage on the right represents the mutation frequency. (**C-H**) Lollipop charts showing the mutation sites on (**C**) MUC16, (**D**) SYNE1, (**E**) OBSCN, (**F**) ZFHX4, (**G**) ABCA13 and (**H**) CSMD1 proteins for high and low patients. (**I**) Risk score differences between patients with WT and MT types of MUC16, SYNE1, OBSCN, ZFHX4, ABCA13 and CSMD1. *WT* wild type, *MT* mutant type.

The top 20 most frequently mutated genes for TCGA patients with high and low risks were depicted by waterfall plots in Fig. 9A and B, respectively. Notably, differences in mutation frequencies reached statistical significance for six genes including MUC16, SYNE1, OBSCN, ZFHX4, ABCA13 and CSMD1, with low risk patients exhibiting higher mutation frequencies (Fig. 9C–H). In addition, risk scores were higher for patients with wild type (WT) of these six genes than that with mutant type (MT) (Fig. 9I). These results consistently correlated low risk patients with increased gene mutation burdens, which also supported the improved responsiveness to ICI treatment for this group of patients.

### Drug sensitivity analysis

In consideration of the non-negligible role of chemotherapies in CRC patients’ comprehensive management strategy, we determined to search the suitable anti-tumor drugs for patients with high and low risk respectively. As shown in Fig. 6B, there were significant differences in the IC50s for 5-flurouracil, cisplatin, sorafenib and erlotinib between high and low risk patients. Low risk patients were demonstrated to respond better towards 5-flurouracil, cisplatin and sorafenib, whereas high risk patients could potentially gain more benefit from erlotinib treatment.

### Model gene expression patterns at single cell resolution

**Figure 10:**
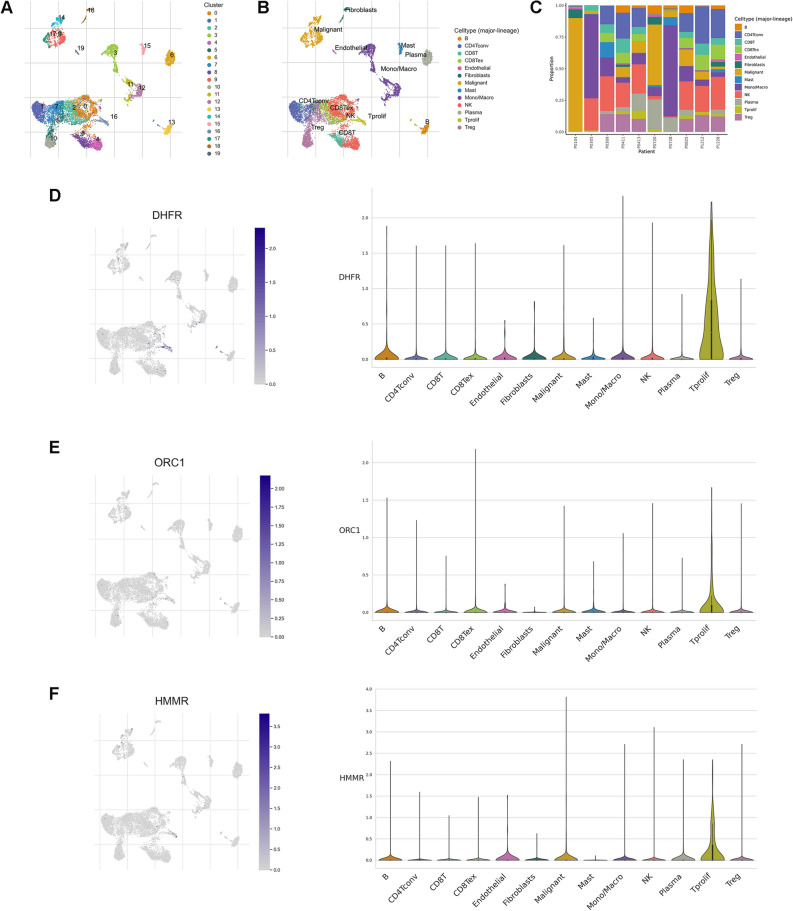
DGRG expression at single cell resolution. (**A**) Identification of different cell clusters. (**B**) Annotation of different cell lineages. (**C**) The percentage of each cell subtype in different patients. (**D-F**) Expression of (**D**) DHFR, (**E**) ORC1 and (**F**) HMMR in different cell lineages.

To decipher the expression patterns of genes involved in DGRG risk score model from the single cell resolution, we selected one single cell RNA-seq dataset GSE146771 from TISCH website. The dataset contains transcriptomes of 10,468 single cells from 10 CRC patients. After quality control and dimension reduction, a total of 20 cell clusters were obtained (Fig. 10A). Further cell type annotation identified 13 major cell lineages (Fig. 10B). It was suggested that DHFR was mainly expressed in proliferative T cells (Fig. 10D). Increased ORC1 and HMMR expressions could also be observed in proliferative T cells (Fig. 10E,F). As the three model genes were all highly expressed in low risk patients, the results to some extent reflected the stronger immunogenicity for low risk patients, which supported the previous result correlating low risk patients with a “hot tumor” phenotype.

## Discussion

Tumor cells rely heavily on glycolysis to meet the heightened energy requirement. Mounting evidence has suggested the pro-tumorigenic role of enhanced glycolysis in the pathogenesis of CRC. In addition to directly fueling tumor cells in promotion of the rapid growth of them, elevated glycolytic activity generates excessive lactate, a widely-acknowledged oncogenic substance. On the one hand, lactate is responsible for increased angiogenic events in TME^16,17^, thus promoting the delivery of nutrients to TME and further enhancing the invasiveness of tumor cells^18^. On the other hand, lactate was also reported to hamper the killing effects on tumors by immune cells and promote tumor cells’ escape from immune response^19,20^. Therefore, glycolysis represents a novel therapeutic target that could be harnessed to alter the malignant behavior of tumor cells. Disulfidptosis is a newly-emergent PCD mode characterized by aberrant disulfide bond formation in cytoskeleton proteins. Interestingly, this PCD mode is supposed to be induced under glucose-depleted conditions and may therefore had intersections with glycolysis. Besides, according to our primary analysis, some disulfidptosis-related genes were differentially expressed between CRC tumor and normal tissues, and showed tight correlations with clinical features, which highlighted the potential role of disulfidptosis in CRC tumorigenesis. Based on this, we further estimated CRC patients’ enrichment scores on disulfidptosis- and glycolysis-related gene sets and found there were positive associations between two types of enrichment scores. We postulated that the glucose-deficient environment caused by elevated glycolysis could increase tumor cells’ susceptibility to disulfidptosis. In support of this, some previous studies also observed decreased glucose concentration in tumor surroundings and attributed it to higher glucose consumptions^15^. Further studies are warranted to reveal the molecular mechanisms underlying the disulfidptosis-glycolysis linkage during tumorigenesis.

Next, WGCNA analysis was performed to identify gene modules showing the strongest correlations with both disulfidptosis and glycolysis activities in TCGA and GEO cohort, respectively. A total of 121 hub genes were obtained through intersection analysis. KEGG and GO analysis correlated these hub genes with cell proliferation, cancer development and metabolic activity. Subsequently, we developed a three gene-based DGRG risk score signature in TCGA cohort through univariate and LASSO Cox regression analysis, which had favorable performance in distinguishing the survival outcomes of CRC patients. The implicative value of DGRG risk score on CRC patients’ prognosis was latter verified in GEO cohort. Moreover, we constructed a nomogram by integrating DGRG risk score and other prognosticators to provide an accurate prediction for 1, 3 and 5 year survival probabilities.

Previous studies have revealed the sophisticated crosstalk between glycolytic activities and the TME landscape of cancer patients. On the one hand, the metabolic reprogramming from oxidative phosphorylation towards glycolysis triggered by CD28-mediated PI3K signaling pathway activation is essential for T lymphocytes to exert the tumor-eliminating effector functions^21^. While some immunosuppressive molucules were reported to impede glycolysis in T lymphocytes, resulting in the functional inhibition of the cells^22^. On the other hand, the end-stage product of glycolysis, lactate, was a well-defined immunosuppressant, which could be targeted to overcome cancer patients’ resistance to immunotherapies^23^. Although the exact implication of disulfidptosis activity on cancer patients’ immune characteristics is still unknown, two disulfidptosis-related gene SLC7A11 and SLC3A2, has been reported to play a role in the immune process during tumorigenesis. The anti-tumor cytokine IFN-γ derived from CD8 T cells could downregulate the expression of SLC7A11 and SLC3A2, which further activate the ferroptosis pathway and exhibit anti-tumor effect^24^. Inhibition of SLC7A11 could enhance the tumor-killing effect of immune cells and sensitize cancer cells’ responsiveness to immunotherapies^25^. Therefore, we came up with the question of whether the current DGRG risk score signature could be used to distinguish the TME landscape and immunotherapeutic sensitivity for CRC patients. To tackle with this, we first estimated the infiltration levels of different immune cells using multiple algorithms. The results revealed that a majority of anti-tumor immune cells were more densely populated in the TME of low risk patients. Next, comparisons of the anti-tumor immune cycle showed that the abilities of cancer antigen presentation, priming and activating immune cells, trafficking immune cells to tumors, recognition and killing of tumor cells were stronger in the low risk group, indicating the sufficiently-activated anti-tumor immune response for low risk patients. In addition, three model genes ORC1, DHFR and HMMR, of which the upregulation was associated with low risk patients, were all found to have up-regulated expressions in proliferative T cell lineages based on single cell RNA-seq analysis. Taken together, it is reasonable to consider CRC patients with low risk level as bearing immune-inflamed “hot” tumors.

Thereafter, we investigated the correlation of DGRG risk score signature with CRC patients’ responsiveness to immunotherapies. First, low risk patients was associated with high immune checkpoint gene expressions, enhanced MSI-H status as well as increased gene mutation burdens, indicating the improved responsiveness to ICI treatment for this group of patients. Next, we applied the DGRG risk score to two external ICI cohorts and found that low risk patients were either correlated with prolonged survival or improved treatment responses. Finally, IHC analysis on 10 CRC samples identified the positive expression correlations of the model gene ORC1 immune checkpoint gene PD-L1. Since ORC1 showed the strongest negative correlation with DGRG risk score among the three model genes, the result also to some extent supported the identification of low risk patients as potential beneficiaries from ICI therapies.

In the present DGRG risk score model, patients with different risk level were correlated with distinct survival outcomes and responsiveness to ICI therapies, which, to some extent, could be attributed to disparities in TME landscapes and biological function. First, low risk patients encompassed a hot tumor phenotype, which were supposed to have advantages in survival and ICI sensitivity^9^. Second, immune cells with anti-tumor properties such as CD8 T cells, DCs, NK cells, CTLs and M1 macrophages were all more densely-infiltrated in the TME of low risk patients. CD8 T cells control tumor growth either by directly killing tumor cells via the cytotoxic activity, or by orchestrating the tumor antigen presentation process^26^. Cancer patients with abundant CD8 T cell infiltration tended to exhibit favorable prognosis^27^ and respond better to immunotherapies^28^. Conversely, dysfunction and exhaustion of CD8 T cells during tumorigenesis was suggestive of progression of cancer and insensitivity to immunotherapy^29^. DCs have been reported to be essential players in anti-cancer immunity^30^. Through direct tumoricidal activity, antigen presentation and the complex interactions with lymphocytes, DCs are supposed to restrict the rapid growth and invasion of tumor cells^31,32^. For CRC patients, the infiltration level and maturation status of DCs were tightly associated survival outcomes and responsiveness to ICI treatment^30,32,33^. More importantly, DC itself represents an optimal candidate for cancer vaccines, which highlights the therapeutic utility of it in anti-cancer immunotherapy^32^. NK cells and CTLs take effects at the end-stage of anti-cancer immunity which are responsible for the ultimate killing and eradication of tumor cells^34,35^. Upregulated infiltrations of these two types of immune cell are associated with improved survival outcomes as well as favorable immunotherapeutic response for CRC patients^36,37^. The pro-inflammatory M1 macrophages are indispensable for the induction of anti-tumor Th1 response. Besides, M1 macrophages directly targeted tumor cells via the secretion of anti-tumor cytokines^38^. In CRC, higher M1 amounts were also associated with prolonged OS^39^. Third, multiple carcinogenic pathways (e.g. TGF beta signaling pathway, Wnt beta catenin pathway)^40–43^ and stromal activities^44^ all had elevated enrichment levels in high risk patients, which could also undermined the anti-cancer immunity and ICI responsiveness.

Finally, we used the “pRRophetic” to screen out suitable medication strategies for high and low risk patients respectively. It was revealed that three commonly-used drugs for malignancies in digestive system: 5-flurouracil, cisplatin and sorafenib all had lower IC50s in low risk patients, whereas erlotinib treatment might be more effective for high risk patients.

Admittedly, the present study had certain limitations. First, we only observed the potential positive associations between disulfidptosis and glycolysis from gene expression level and explain the potential reason by literature review, which requires to be supported by more experimental evidence. Second, as an in silico study conducted by retrospectively analyzing public datasets, the prediction for survival and ICI therapeutic responsiveness needs to be validated in real-world prospective CRC patient populations. Third, since the conceptualization of disulfidpotosis is in its infancy, future newly-emergent marker genes are still needed to be enrolled to increase the predictive performance of the current risk score model. Nevertheless, this to our best knowledge was the first study to explore the crosstalk of disulfidptosis- and glycolysis- related genes in the pathogenesis of CRC, establish a relevant prognostic model and analyze the impact of it on the TME landscape and therapeutic responsiveness.

In summary, our present work used WGCNA method to identify disulfidptosis- and glycolysis-related genes and developed a DGRG risk score signature to quantify CRC patients’ risk level. There were significant differences in clinical features, immune infiltrations, functional enrichment patterns and gene mutation landscapes for patients with high and low risk levels. More importantly, the DGRG risk score signature had favorable performance in predicting the survival outcomes and determining the ICI therapeutic sensitivity for CRC patients, which will provide novel insights into the development of personalized treatment strategies and promote the future research and development of novel medications.

## Methods

### Data collection

The analytical process of the study was depicted in Fig. S1. We downloaded gene expression and clinical profiles for COAD and READ patients from The Cancer Genome Atlas (TCGA) database. In addition, GSE39582, a microarray dataset for CRC patients were obtained from the Gene Expression Omnibus (GEO). After excluding the patients with unknown follow-up information, there were 515 and 556 cases being selected for further analysis for TCGA and GSE39582, respectively. Baseline information for the enrolled patients were listed in Table S1.

To make sure the gene expression data was comparable, the FPKM format of TCGA RNA-seq data was transformed into TPM format, which was believed to resemble GEO microarray data. Besides, the “Combat” algorithm of R package “sva” was used to eliminate the batch effect within the two cohorts.

### Gene set preparation and ssGESA analysis

A disulfidptosis gene set containing 22 genes were curated from related articles. Two Glycolysis-related gene sets: HALLMARK_GLYCOLYSIS and REACTOME_GLYCOLYSIS were downloaded from MSigDb website (Table S2). We estimated enrichment scores of the gene sets for CRC patients using single sample gene set enrichment analysis (ssGSEA) method. The calculation was performed by “GSVA” package in R.

### WGCNA analysis

Based on previously described protocols^45^, we conducted WGCNA analysis to identify hub genes showing the strongest correlations with both disulfidptosis and glycolysis activities. First, we selected genes with top 5000 of median absolute deviation (MAD) for TCGA and GEO cohort respectively for hierarchical clustering analysis. Pearson correlation coefficients were used to evaluate the distance between each gene. Then, we identified the soft thresholding power (β) value based on the scale-free topology network criterion and converted the expression matrix into a adjacency matrix. Next, according to topological overlap measure (TOM) and dissimilarity (1-TOM), the adjacency matrix was further clustered and formed into a topology matrix. A dynamic tree cut algorithm was employed for gene module determination. The minimum gene number in each module was set as 30. Finally, the correlations of the eigengenes in each module with the disulfidptosis and glycolysis activities were estimated by Pearson correlation analysis. The most correlated module was selected as the hub gene modules and the composing genes were defined as hub genes. Kyoto Encyclopedia of Genes and Genomes (KEGG) database contains high-level function profiles for target genes^46^. Gene otology (GO) analysis was also a commonly-used bioinformatic tool that could provide biological function information for genes of interests based on three dimensions: molecular function (MF), biological process (BP), and cellular component (CC). Herein, we conducted KEGG and GO pathway enrichment analysis using R package “clusterprofiler”^47^.

### Construction and validation of DGRG risk score signature

In an attempt to quantify CRC patients’ risk level, we developed a DGRG risk score signature. In brief, DGRGs were first subject to univariate Cox regression analysis to evaluate the prognostic value. Then we conducted least absolute and selection operator (LASSO) regression analysis using R package “glmnet” to further reduce overfitting. The formula for DGRG risk score calculation was: $\mathrm{DGRG Risk score}=\sum (Exp i*Coef i)$, with Exp i and Coef i indicating the expression level and multivariate Cox coefficient of each model gene, respectively. We used TCGA cohort for model construction and validated it in GEO cohort. Principle component analysis (PCA) was conducted to verify the robustness of risk level classification. Besides, log-rank test with Kaplan–Meier plot were used to assess disparities in overall survival (OS) between high and low risk patients.

### Nomogram construction

To augment the clinical application value of DGRG risk score signature, we first conducted univariate and multivariate Cox regression analysis integrating risk score and other clinical factors. Elements with statistical significance were further used to construct a nomogram, which was visualized by “rms” package in R. Calibration curves were drawn to evaluate the consistency between actual survival and that predicted by nomogram. Besides, we performed receiver operating curve (ROC) analysis and used the area under curve (AUC) values to assess the efficacy of nomogram in predicting patients’ OS at different time points.

### Functional enrichment analysis

To gain an insight into the biological functions correlated with high and low risk patients respectively, we performed gene set enrichment analysis (GSEA) analysis based on KEGG gene set and gene set enrichment analysis (GSVA) analysis based on Hallmark gene set. Besides, using ssGSEA method, we quantified the enrichment scores of immune and stromal-related gene signatures developed by Mariathasan et al.^41^ (Table S2), as well as the metabolism-related gene signatures developed by Yang et al.^48^, and latter compared the differences in enrichment scores between high and low risk patients.

### Immune infiltration and anti-tumor immunity analysis

We used TIMER, MCPcounter and QUANTISEQ algorithms to estimate the abundance of various immune cells infiltrated in TME. The calculation process was conducted by R package “IOBR”^49^. A previous research conceptualized the anti-cancer immune response into a seven-step cyclic event^50^. Herein, regulatory genes involved in each step were obtained from the tracking tumor immunophenotype (TIP) website for ssGSEA analysis^51–53^. Differences in immune cell infiltration levels and anti-tumor immunity activities between high and low risk patients were then compared.

### ICI therapeutic response analysis

To assess the potential utility of DGRG risk score in guiding ICI therapy, we compared the expression differences of PD1, PDL1 and CTLA4 between high and low risk patients, which are three commonly-used targets of ICI therapies. In addition, tumor mutation burden (TMB) and the microsatellite instability (MSI) status were recognized as two metrices to assess ICI therapeutic responses, with higher TMB value^54–56^ and MSI-high (MSI-H) status^57,58^ demonstrating elevated sensitivity to ICI treatment. Herein, we estimated TMB values in Perl software using annotation files^59^. Besides, MSI profiles for CRC patients were obtained from TCGA website. Disparities of TMB and MSI between the two risk groups were subsequently compared. Moreover, the DGRG risk score was applied to two external ICI cohorts to verify the predictive efficacy. The cohorts include IMvigor210 (urothelial carcinoma patients receiving anti-PD-L1 treatment) and David Liu’s cohort (melanoma patients receiving anti-PD1 treatment)^60^.

### Gene mutation analysis

Gene mutation data for CRC patients was downloaded from TCGA database. The top 20 genes with the highest mutation frequencies were identified for two risk groups respectively and visualized in the form of waterfall plot via “maftools” packages in R^61^.

### Drug sensitivity analysis

The “pRRophetic” R package used empirical Bayesian approach and ridge regression methodology with tenfold cross-validation to fit transcriptome data of tissue samples with the experiment-based drug sensitivity profiles of multiple cancer cell lines from GDSC database^62^. Herein, we utilized this package to estimate the half inhibitory concentration (IC50) values for 5-flurouracil, cisplatin, sorafenib and erlotinib, in order to select the suitable anti-tumor medication for CRC patients at different risk levels.

### DGRGs expression at single cell resolution

Tumor Immune Single Cell Hub (TISCH) was a large-scale online database that integrates single-cell transcriptomic profiles of nearly 2 million cells from 76 high-quality tumor datasets across 27 cancer types^63^. Herein, we utilized this database to unearth the expression patterns of DGRGs in different cell subtypes in the TME of CRC patients.

### Tissue collection and immunohistochemistry

We conducted this study according to the Declaration of Helsinki. A total of 10 fresh CRC tumor tissues were obtained during surgical removal in Renmin hospital of Wuhan University to prepare into formalin-fixed, paraffin-embedded (FFPE) specimens. The whole study design was approved by the Medical Ethics Committee of Renmin Hospital of Wuhan University (WDRY2021-K126), and all participants have signed written informed consent. Immunohistochemical (IHC) staining of FFPE specimens were performed following standardized procedures as previously described^64^. The primary antibodies used for IHC experiment included anti-ORC1 (Cohesion bioscience, CPA1841) and anti-PD-L1 (Servicebio, GB11339A) antibodies. IHC profiler plugin of Image J software was used to estimate the optical density score of each sample based on previously-described methods^65^.

### Statistical analysis

Comparisons of quantitative and qualitative data used Wilcoxon rank sum test and Chi-square test, respectively. For survival data, log-rank test was chosen and the result was displayed in the form of Kaplan–Meier plots. Besides, univariate and multivariate Cox regression analysis were used to assess the independent prognostic value of factors. The results were demonstrated in the form of forest plots. All statistical analyses were performed in R software (version 4.1.2) and SPSS Statistics 25 software. Statistical significance was set at p < 0.05.

### Ethics declarations

Research involving human research participants must have been performed in accordance with the Declaration of Helsinki. The study design was approved by the Medical Ethics Committee of Renmin Hospital of Wuhan university (WDRY2021-K126). Written informed consent has been provided by all participants.

### Supplementary Information

Supplementary Figures.

Supplementary Tables.

## Data Availability

The datasets supporting the conclusions of this article are available in the Cancer Genome Atlas (https://portal.gdc.cancer.gov, COAD and READ category) and Gene Expression Omnibus (https://www.ncbi.nlm.nih.gov/geo, accession ID: GSE39582).
