# Supplementary material for: A novel disulfidptosis and glycolysis related risk score signature for prediction of prognosis and ICI therapeutic responsiveness in colorectal cancer
Source: Sci Rep. 2023 Aug 16;13:13344. doi: 10.1038/s41598-023-40381-5 (PMC10432503; doi:10.1038/s41598-023-40381-5)
Supplement: Supplementary file 1 — Supplementary Figures. [file 41598_2023_40381_MOESM1_ESM.docx]

**A novel disulfidptosis and glycolysis related risk score signature for prediction of prognosis and ICI therapeutic responsiveness in colorectal cancer**

Jiazheng Li^1^, Chao Yang^1^ and Yongbin Zheng^1*^

^1^ Department of Gastrointestinal Surgery, Renmin Hospital of Wuhan University, Wuhan, China

^*^Correspondence: Yongbin Zheng; Email: yongbinzheng@whu.edu.cn


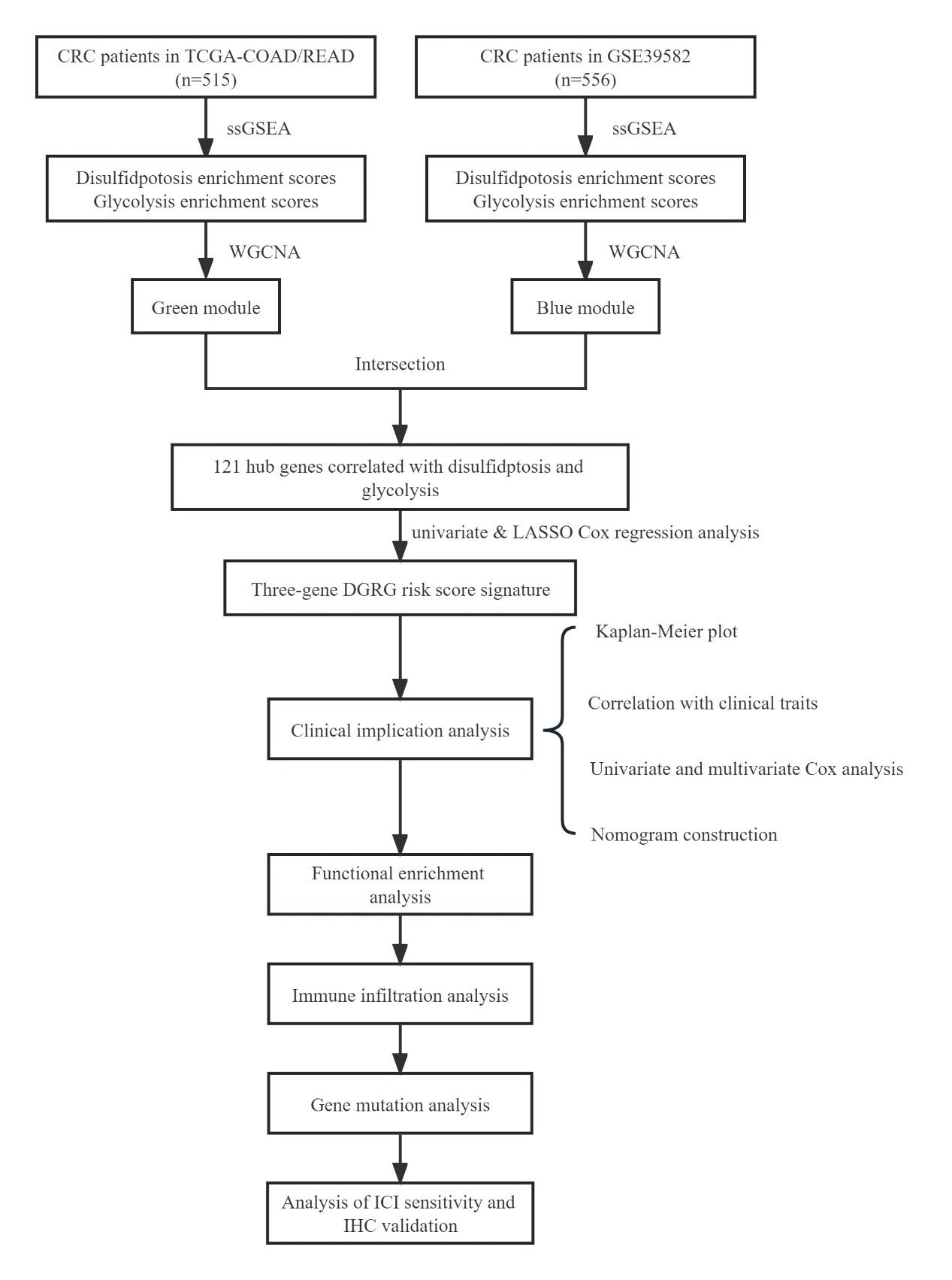


Fig. S1 Work flow of the study.


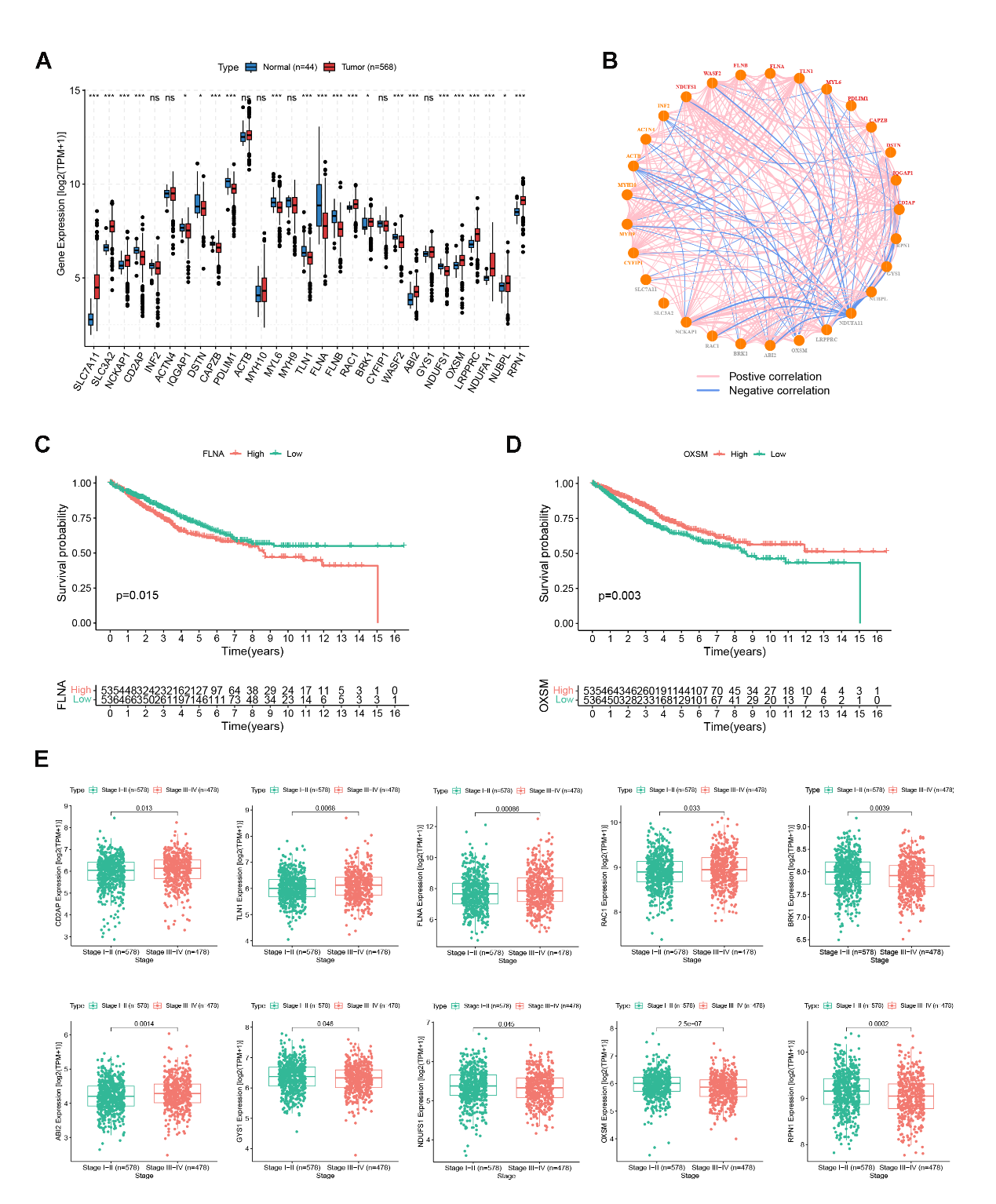


Fig. S2 Clinical relevance of 29 disulfidptosis-related genes. **A** Differential expression analysis of disulfidptosis-related genes between tumor and normal tissues in TCGA cohort. **B** Pearson correlation analysis for 29 disulfidptosis-related genes. The thickness of the lines represent the correlation strength. **C, D** Kaplan-Meier plots for patients with different expressions of **(C)** FLNA and **(D)** OXSM. **E** Differential expression analysis for CD2AP, TLN1, FLNA, RAC1, BRK1, ABI2, GYS1, NDUFS1, OXSM and RPN1 between patients with stage I-II and stage III-IV tumors.


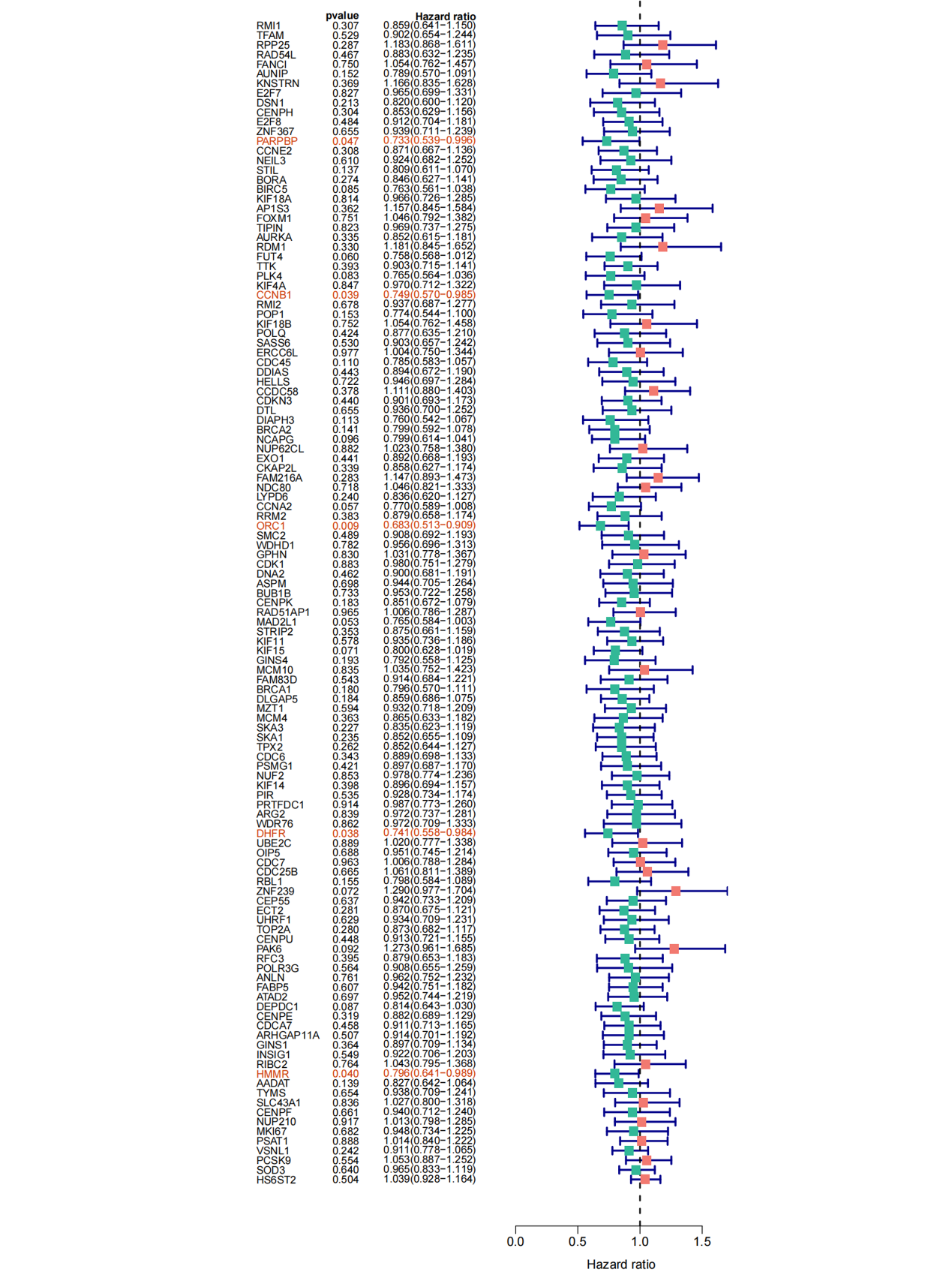


Fig. S3 Univariate Cox regression analysis for 121 hub genes.


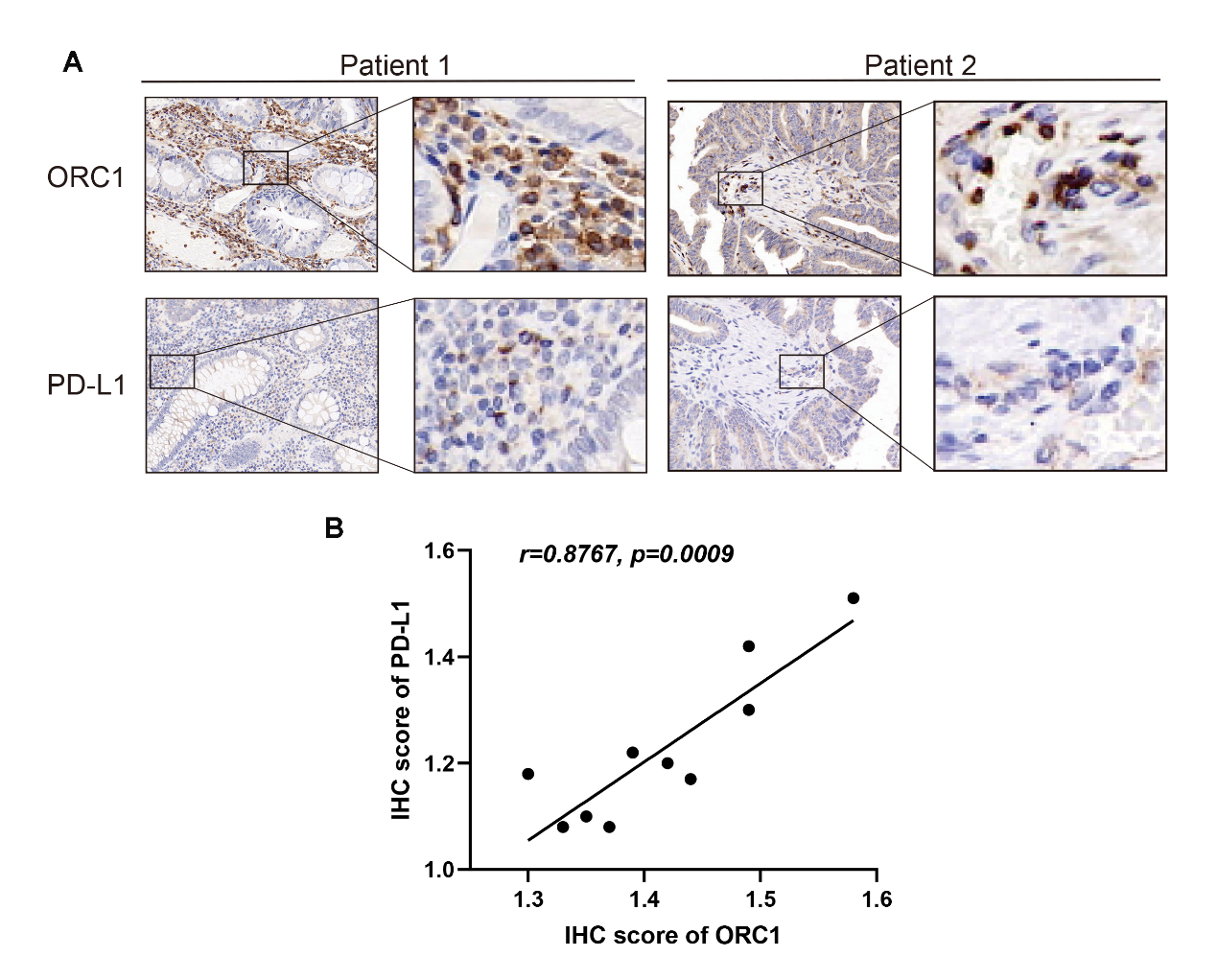


Fig. S4 Validation of TME landscape by IHC experiment. **A** Representative IHC image from two patients. **B** Scatter plot showing the expression correlations of ORC1 with PD-L1.
